# Comparison of Pore Structures of Cellulose-Based Activated Carbon Fibers and Their Applications for Electrode Materials

**DOI:** 10.3390/ijms23073680

**Published:** 2022-03-27

**Authors:** Ju-Hwan Kim, Sang-Chul Jung, Hye-Min Lee, Byung-Joo Kim

**Affiliations:** 1 Convergence Research Division, Korea Carbon Industry Promotion Agency (KCARBON), Jeonju 54853, Korea; bear9601@kcarbon.or.kr; 2 Department of Environmental Engineering, Sunchon National University, Sunchon 57922, Korea; jsc@sunchonm.ac.kr; 3 Department of Carbon-Nanomaterials Engineering, Jeonju University, Jeonju 55069, Korea

**Keywords:** cellulose fiber, activated carbon fiber, steam activation, electric double-layer capacitor (EDLC)

## Abstract

This study presents the first investigation of cellulose-based activated carbon fibers (RACFs) prepared as electrode materials for the electric double-layer capacitor (EDLC) in lieu of activated carbon, to determine its efficacy as a low-cost, environmentally friendly enhancement alternative to nanocarbon materials. The RACFs were prepared by steam activation and their textural properties were studied by Brunauer–Emmett–Teller and non-localized density functional theory equations with N_2_/77K adsorption isotherms. The crystallite structure of the RACFs was observed by X-ray diffraction. The RACFs were applied as an electrode material for an EDLC and compared with commercial activated carbon (YP-50F). The electrochemical performance of the EDLC was analyzed using galvanostatic charge/discharge curves, cyclic voltammetry, and electrochemical impedance spectroscopy. The results show that the texture properties of the activated carbon fibers were influenced by the activation time. Crucially, the specific surface area, total pore volume, and mesopore volume ratio of the RACF with a 70-min activation time (RACF-70) were 2150 m^2^/g, 1.03 cm^3^/g and 31.1%, respectively. Further, electrochemical performance analysis found that the specific capacitance of RACF-70 increased from 82.6 to 103.6 F/g (at 2 mA/cm^2^). The overall high specific capacitance and low resistance of the RACFs were probably influenced by the pore structure that developed outstanding impedance properties. The results of this work demonstrate that RACFs have promising application value as performance enhancing EDLC electrode materials.

## 1. Introduction

The electric double-layer capacitor (EDLC) is an energy storage device that has attracted recent attention along with the lithium-ion battery. The mechanism of energy storage for the ELDC relies on the physical adsorption and desorption reaction of ions on the electrode surface [1,2]. The scope of application of the EDLC has recently extended within the fields of energy (new renewable energy development and smart grids) and transportation (electric and hybrid cars) based on the rapid charge-discharge rate due to high output density despite low energy density and semi-permanent lifetime [3,4,5]. The electrochemical properties of the EDLC are determined by three factors: the electrolyte [6], current collector [7], and active electrode material [8], the last of which has the greatest influence. Thus, the active electrode material of the EDLC should have a large specific surface area and outstanding electrical conductivity to facilitate the storage of a large number of ions. For this reason, porous carbon materials have been widely used. Notably, among the various porous carbon materials for the EDLC, activated carbon (AC) shows numerous advantages from outstanding electrical conductivity to physical and chemical stability, large specific surface area, and low cost, such that it has been used as the main active electrode material for the EDLC.

With the recent extensions of the scope of EDLC application, studies to enhance the performance of the EDLC have been conducted regarding the use of nanocarbon materials with high electrical conductivity (e.g., carbon nanotubes [9,10,11], graphene [11,12,13,14]) as an active electrode material, or the control of pore structure for improving the ion diffusion resistance [15,16]. However, the EDLC applications proposed in these studies are not environmentally friendly and have high costs. Moreover, they tend to involve a complex process of material synthesis; hence, their feasibility is low in an actual industrial setting.

Activated carbon fiber (ACF) is a fiber form of AC that serves as a highly desirable active electrode material for the EDLC due to high specific surface area and outstanding electrical conductivity [16,17,18,19,20,21]. In particular, ACF is distinguished from AC by having a micropore structure that is oriented along the fiber axis with the hexagonal carbon layers of well-organized ribbon or sheet conformation [22]. This pore structure of ACF is known to allow a higher adsorption amount and rate than AC through direct external exposure of micropores [23,24]. Gan, et al. reported the short ion diffusion pathways due to the well-defined surface pores of activated carbon fibers led to an excellent electrochemical performance [25]. Hence, as ACF allows a reduction in ion diffusion resistance compared to AC, it is a highly desirable electrode material that can improve the Warburg impedance of the EDLC.

The production of ACF uses such precursors as polyacrylonitrile (PAN) fiber [18], pitch fiber [19,24], and cellulose fiber [20,21]. Notably, rayon fibers among cellulose fibers comprise high-purity cellulose to exhibit high crystallinity, which leads to high electrical conductivity upon its use in the production of carbon fibers or ACFs and has the advantage of fluent control of the pore structure [20,26]. In addition, cellulose derived ACF produced from wood pulp offers a vision for manufacturing high-performance porous materials from renewable resources [27]. In addition, rayon-based ACF (RACF) has greater hardness than pitch-based ACFs, such that less powder is formed to allow easier processing, while the low swelling of crystal grains based on the migration of electrolyte ions during the charge-discharge process poses an additional advantage [19].

In this study, RACF with outstanding pore characteristics and crystallinity was used in lieu of AC as the active electrode material to enhance the performance of an EDLC. The steam activation method was used to produce RACF through a low-cost and environmentally friendly process. The pore development behavior with an increase in activation time was investigated through comparative analysis of textural properties and micropore structures. The performance of the EDLC was analyzed using RACF as the active electrode material of the coin cell. A detailed analysis was performed on the correlation between cell performance and pore structure for RACF in comparison with YP-50F.

## 2. Results and Discussion

### 2.1. X-ray Diffraction Analysis

**Figure 1:**
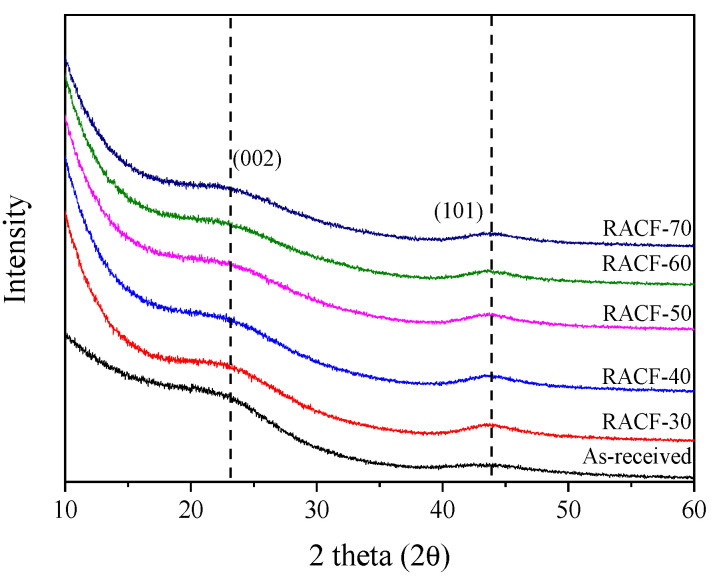
XRD patterns of RACF as a function of various steam activation conditions.

XRD is a highly useful method to analyze the changes in crystal structure in accordance with ACF pore development. Figure 1 shows the XRD curves for the RACF produced under various activation conditions. The XRD curves for the RACFs appeared as that of a typical isotropic carbon material with a C(002) peak at 23° and a C(101) peak at 43°, while peaks of other ashes were not detected [28]. In addition, the intensity of the XRD curves decreased as the activation time increased due to the oxidation of crystal grains. All (002) or (101) peak centers of the RACFs appeared to be consistent irrespective of the activation time.

**Figure 2:**
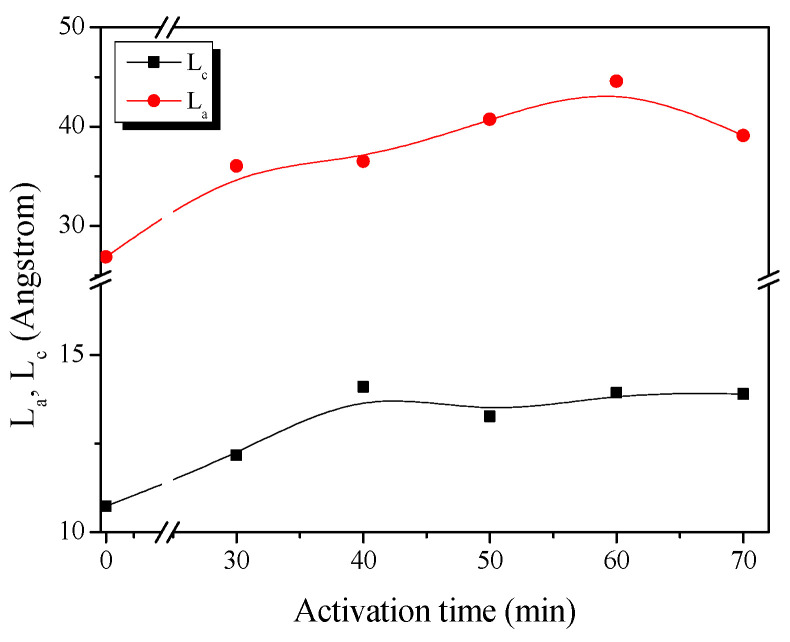
Structural characteristics of RACF as a function of various steam activation conditions.

Figure 2 shows the changes in the L_c_ (crystallite height) and L_a_ (crystallite size) of RACF as a function of the activation time calculated from the XRD curve. The XRD illustrates well the changes in the crystal structure of the RACF based on activation time as statistical data. In other words, as the activation process occurred under a constant temperature, rather than indicating the crystal grain growth for RACF, the increases in L_a_ and L_c_ indicate the relative increase in crystal grain size due to noncrystalline oxidation. On the other hand, the decrease in L_a_ and L_c_ indicates the oxidation of the crystal grain edge.

In the graphite crystalline structure, the layers of the (002) plane consist of strong hybridized sp^2^ bonds, and the vertical π bond on the (002) plane results in weak interlayer bonds. Crystalline changes were thus more easily observed for the (002) plane than the (101) plane [29,30].

The evolution of the RACF can be categorized into three steps based on the changes in L_c_ and L_a_ as follows. Step 1 (RACF-30 to -40): the L_c_ and L_a_ of the RACF increased from 10.7 to 14.1 Å and from 26.9 to 36.5 Å, respectively, as the activation time increased. Step 2 (RACF-50 to -60): the L_c_ of the RACF decreased slightly from 14.1 to 13.9 Å but the L_a_ increased from 36.5 to 44.6 Å. Step 3 (RACF-70): the L_c_ of the RACF was maintained at 13.9 Å but the L_a_ decreased to 39.1 Å. Hence, the relative increase in L_c_ and L_a_ is presumed to have been caused by preferential amorphous oxidation in Step 1 through the activation mechanism. As amorphous oxidation continued in Step 2, the L_a_ increased, while the simultaneous oxidation of the crystal grain edge led to a slight decrease in L_c_. In Step 3, the L_a_ decreased presumably as the oxidation occurred only on the crystal grain edge. Overall, it can be understood that, with an increase in the activation time, the crystal favors amorphous oxidation, and the oxidation on the crystal grain edge occurs subsequently.

### 2.2. Adsorption Isotherm and Textural Properties

**Figure 3:**
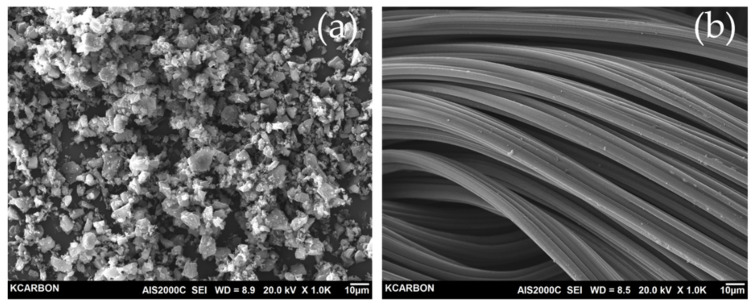
SEM images of the electrode materials (**a**) YP-50F, (**b**) RACF.

The three-dimensional shape and surface texture of the samples have been characterized by SEM. As shown in Figure 3, the YP-50F and RACF were observed as granular and fibrous, respectively. For the RACF, the surface was typically very smooth, without any macroscopic defects. It can be seen that the fibrous structure of the RACF is not destroyed because the amorphous is mainly oxidized in the process of physical activation by steam.

**Figure 4:**
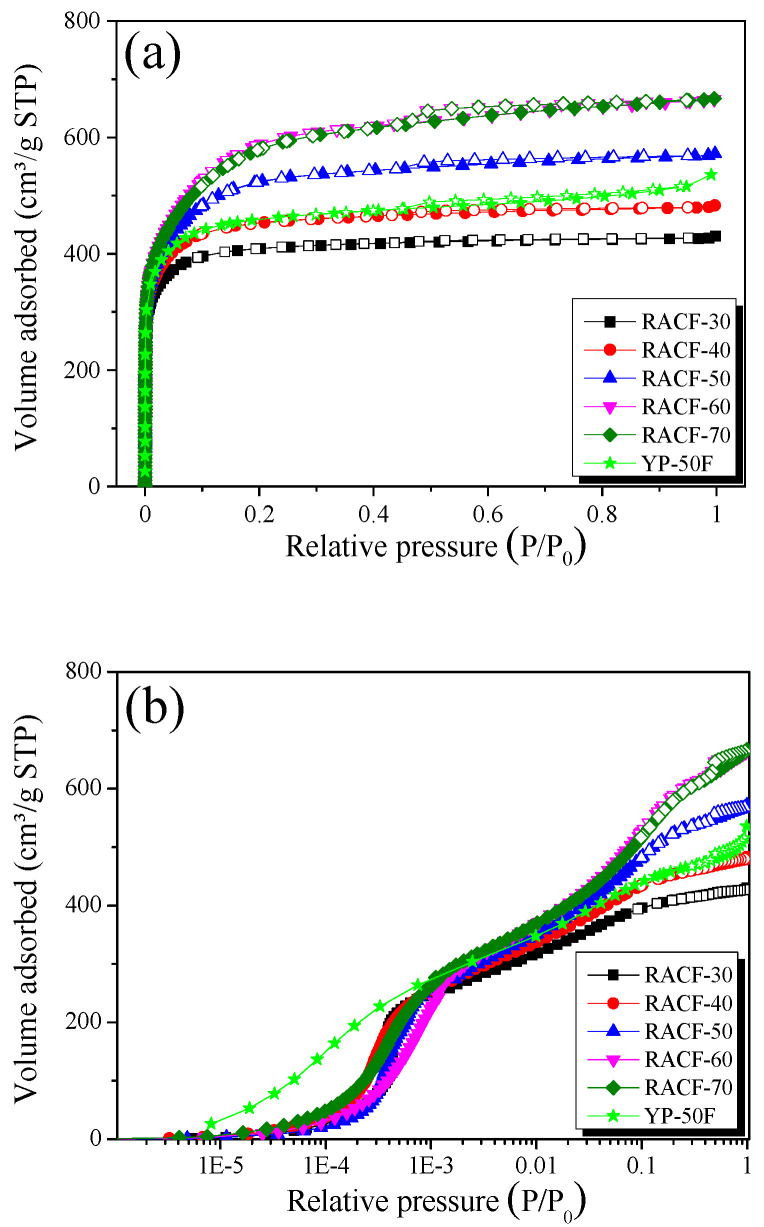
N_2_/77K isotherm adsorption-desorption curves of RACF as a function of various activation conditions; (**a**) normal and (**b**) logarithmic.

The N_2_/77K isotherm adsorption-desorption curve is the most common method of analyzing the pore characteristics of porous carbons, thus it was used to examine the changes in the pore characteristics of the RACF based on activation time. Figure 4 shows the N_2_/77K adsorption-desorption curve for the RACF as a function of activation time.

As shown in Figure 4a, the isotherm adsorption curves of the RACF samples uniformly display the Type I curve of the International Union of Pure and Applied Chemistry (IUPAC) classifications [31]. The point of inflection referred to as “the knee” of the curve that appears in the early part of the isotherm curve is due to the monolayer-multilayer adsorption. This indicates the step where monolayer adsorption of N_2_ molecules occurs on the wall, followed by the onset of multilayer adsorption [32]. Hence, the Type I isotherm adsorption curves are displayed by the materials that have a wider range of PSD including microporous materials with narrow micropores or wider micropores and possibly narrow mesopores, depending on the curvature or the relative pressure at the knee [32,33]. In Figure 4a, the isotherm adsorption curves for RACF-30 and -40 each show a knee with low curvature, whereas the isotherm adsorption curves for RACF-50 to -70 each show an increase in knee curvature as the activation time increases. Thus, the RACF samples in Step 1 (RACF-30 and -40) are mainly characterized by micropore development, and those in Steps 2 and 3 (RACF-50, -60, and -70) are characterized by simultaneous development of micropores and mesopores.

The logarithmic scale isotherm adsorption curve (Figure 4b) is a useful method to examine the micropore development behaviors in porous carbons because, for relative pressures below 0.01, it clearly displays the changes in adsorption as a function of the relative pressure. In Figure 4b, all isotherm adsorption curves of the RACF samples show an increase in adsorption at similar pressure domains. YP-50F, however, shows an increase in adsorption in a lower range of relative pressure (1.0 × 10^−5^ to 3.0 × 10^−4^ P/P_0_) than the RACF. Hence, all the RACF samples are presumed to have a PSD of micropores with similar pore diameters, while YP-50F has that with smaller pore diameters. The reason they exhibit varying micropore size distributions despite being produced through identical steam activation processes is possibly due to the differences in crystal structure between rayon fiber and coconut shell as the precursors of RACF and YP-50F, respectively.

The shapes of hysteresis loops have often been correlated to specific pore morphologies [31,33]. All isotherm adsorption curves of the RACF samples showed H4-type hysteresis of the IUPAC classifications [32], while the area of hysteresis increased as the activation time increased. In addition, the same IUPAC H4-type hysteresis was observed for YP-50F as in the RACF. Hence, it is presumed that the RACF and YP-50F share identical slit-shaped pores.

Table 1 presents the textural properties of the RACF samples. The specific surface area and mesopore volume of the RACF from 30 to 60 min activation time increased from 1600 to 2200 m^2^/g and from 0.07 to 0.29 cm^3^/g, respectively. Next, as the activation time increased to 70 min, the specific surface area and micropore volume decreased to 2150 m^2^/g and 0.71 cm^3^ from the peak at 60 min, respectively, while the mesopore volume increased to 0.32 cm^3^/g, exhibiting the highest mesopore volume fraction (R_Meso_ = 31.1%).

In porous carbons, the pores are formed upon crystal grain oxidation during the activation process such that the pore characteristics and crystal structure show a close correlation [29,30]. Notably, in a prior investigation, the edge oxidation of amorphous and crystal grain was shown to have an influence on the development of micropores and mesopores, respectively, through the activation mechanism [30]. In the XRD analysis of this study, the changes in RACF crystal structure could be categorized into three steps depending on the behavior of amorphous or crystal grain edge oxidation, which was compared with the textural properties. In Step 1, the micropore volume was presumed to have increased through amorphous oxidation for RACF-30 and -40. In Step 2, the micropore and mesopore volumes both increased presumably due to the simultaneous edge oxidation of amorphous and crystal grain for RACF-50 and -60. In Step 3, the micropore volume decreased and mesopore volume increased presumably because the edge oxidation occurred mainly on the crystal grains rather than on the amorphous grains for RACF-70.

**Figure 5:**
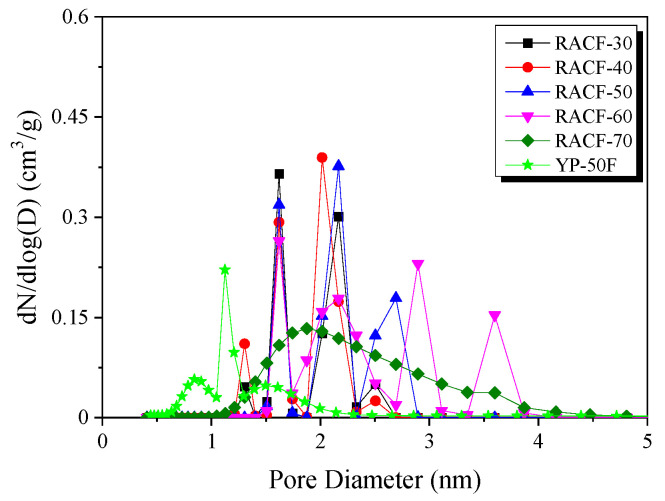
Pore size distribution of RACF as a function of various steam activation conditions according to the NLDFT equation.

The NLDFT method is the most effective method of analyzing the PSD of ACF. Figure 5 shows the PSD curve for the RACF as a function of activation time. In the PSD curves for the RACFs, as in the case of textural properties, three steps of pore development behavior were observed.

In Step 1, RACF exhibited the distribution of narrow micropores and sub-mesopores with diameters of 1.6 and 2.1 nm, respectively. As the activation time increased, the 1.6 nm pores did not vary significantly in volume, but the 2.1 nm pores showed an increase in volume. It is thought that the increase in pore volume in porous carbons due to physical activation occurs through two mechanisms: the pore drilling (leading to the increase in pore diameter) and pore deepening (having no influence on pore diameter) [34]. Hence, the amorphous oxidation in Step 1 is presumed to have increased the pore volume through pore deepening without influencing the pore diameter. In addition, amorphous oxidation is likely to have mainly influenced the increase in the volume of the 2.1 nm pores.

In Step 2, RACF showed changes in PSD in the range of 2–4 nm diameter as the activation time increased, while the volume of the 1.6 nm pores was maintained. At 50 min activation time, both the 1.6 and 2.1 nm pores had maintained their volume, while new pores of 2.7 nm diameter were formed. Next, at 60 min activation time, the PSD curve for sub-mesopores changed from narrow to broad in the range of 2–2.6 nm diameter, along with the PSD of newly formed 2.9 and 3.7 nm mesopores. Hence, the increase in pore diameter and volume with the increase in activation time is likely due to the pore drilling caused by crystal grain edge oxidation. In addition, the continuous increase in the volume of the 2.1 nm pores is thought to be due to the continuous pore deepening caused by amorphous oxidation.

In Step 3, the PSD curve for RACF was considerably broad, ranging from 1–4 nm pore diameter, in contrast to the previous result. In other words, as the activation time increased, the oxidation is thought to have occurred mainly on the crystal grain edge such that the pore volume increased due to pore drilling, and consequently, the micropore volume decreased and the mesopore volume increased.

As shown in Table 1, similar textural properties were observed for RACF-40 and YP-50F. However, the PSD curve for RACF-40 was broader than that for YP-50F, as shown in Figure 5, which coincides with the isotherm adsorption curve shown in Figure 4b, indicating the increase in adsorption for RACF-40 at a higher relative pressure than that for YP-50F. This is presumed to be due to the differences in the crystal structures of the precursors rather than the differences in the unique pore structures of ACF and AC. According to Buchmeiser et al., cellulose fibers have high crystallinity with crystal grains aligned in a highly oriented form along the fiber axis [26]. In other words, while both RACF-40 and YP-50F share the same cellulose-based precursor, the high crystallinity of RACF-40 is presumed to have led to the PSD with larger pore diameters.

### 2.3. Electrochemical Performance

The electrochemical properties of the RACFs were assessed using a 1 M TEBF_4_/PC electrolyte through the GCD curve, CV, and EIS.

**Figure 6:**
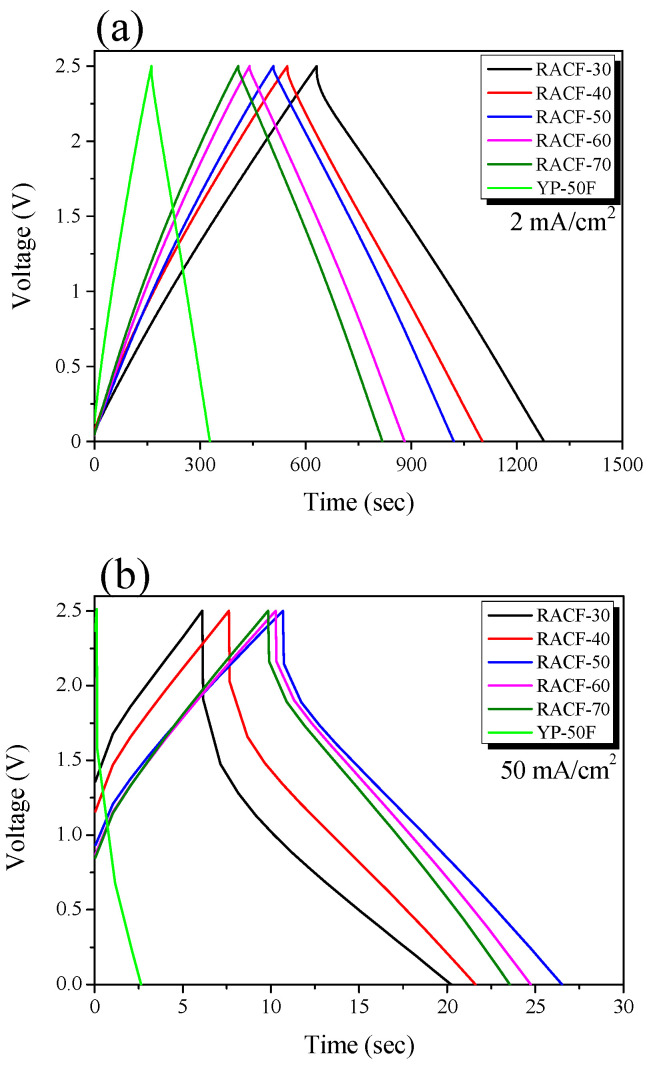
GCD curves of RACF as a function of various steam activation conditions with current densities of: (**a**) 2 mA/cm^2^, (**b**) 50 mA/cm^2^.

Figure 6 shows the GCD curves of the RACFs measured at the current density of 2 and 50 mA/cm^2^. The linear voltage-time dependence demonstrates the typical capacitive behavior of an EDLC. The GCD curves of the RACF samples at 2 mA/cm^2^ current density shows an instantaneous voltage drop (IR drop) of a small value as the charge and discharge curves were in symmetry. In Figure 6b, in contrast, the IR drop significantly increased with an increase in the ohmic resistance of the system under the influence of the increase in current density to 50 mA/cm^2^, which led to the asymmetry of the charge and discharge curves. The magnitude of the IR drop was shown to decrease as the activation time increased at all current density values in the following order: RACF-30 > RACF-40 > RACF-50 > RACF-60 > RACF-70. This IR drop is thought to be due to ohmic resistance of the system and inner resistance of ion diffusion in the nanopores [35]. As shown in Figure 5, RACF pores were found to have increased in volume and diameter as the activation time increased. Thus, for RACF, the inner resistance of ion diffusion in nanopores is thought to have decreased as the activation time increased. Consequently, the lowest inner resistance is predicted for RACF-70, which has the longest activation time.

The IR drop for YP-50F was similar to that of RACF-30 at 2 mA/cm^2^ current density, but larger than any of the RACFs at 50 mA/cm^2^. The sizes of the electrolyte (1 M (C_2_H_5_)_4_NBF_4_) cation and anion were 0.67 and 0.48 nm, respectively, while the sizes of solvated ions have been reported as 1.34 and 1.44 nm, respectively, in previous research [36]. In Figure 5, the PSD of YP-50F showed smaller pore diameters than either RACF-30 or -40. In particular, most pores were distributed at diameters smaller than those of the solvated ions. In addition, the ion diffusion rate for RACF is likely to be faster than YP-50F as an AC, based on the unique pore structure of ACF. Thus, YP-50F is predicted to show higher inner resistance of ion diffusion in nanopores than RACF-30 or -40, consequently showing the largest IR-drop.

**Figure 7:**
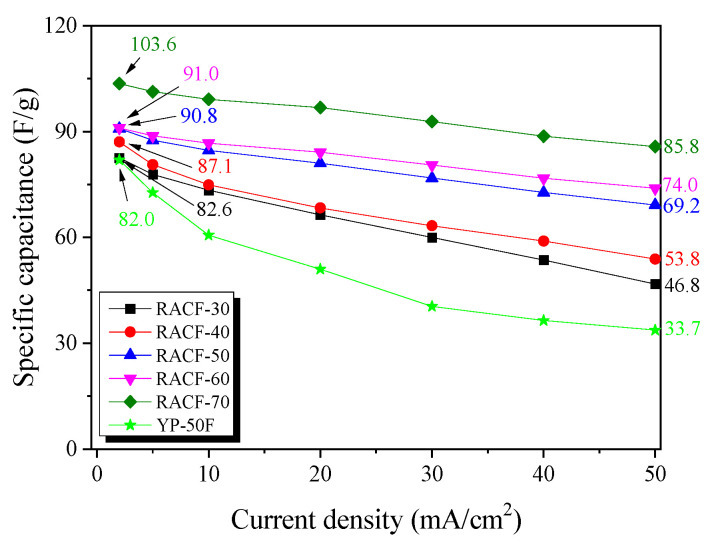
Specific capacitance of RACF as a function of discharge current density.

Figure 7 shows the correlation between current density and specific capacitance. For all the RACF samples, the specific capacitance decreased as the ohmic resistance of system and inner resistance of ion diffusion in nanopores increased with the increase in current density, as was the case shown in Figure 7. As the activation time increased, the specific capacitance of the RACF increased to the range of 82.6–103.6 F/g at the current density of 2 mA/cm^2^ and 46.8–85.8 F/g at 50 mA/cm^2^. For YP-50F, the specific capacitance at 2 mA/cm^2^ was shown to be similar to that of RACF-30, but as the current density increased, the specific capacitance fell to a lower level than that of RACF-30. In addition, the rate of decrease in specific capacitance was substantially higher for YP-50F than for any of the RACF samples. In a prior investigation, the specific capacitance was found to be mainly influenced not by the ion storage based on the volume of micropores of similar size to the solvated ions at a low charge rate, but by the ion diffusion resistance (Warburg impedance) based on the mesopores with larger diameters than the solvated ions at a high charge rate [37]. In this study, likewise, the development and volume of micropores of 1.5–2 nm diameters of similar size to the solvated ions are likely to have influenced the specific capacitance of RACF at low current density, whereas at high current density, the influence is thought to have come from the increase in the mesopore ratio. Thus, due to the high specific surface area (2150 m^2^/g) and mesopore volume ratio (31.1%), the specific capacitance of RACF-70 is presumed to have been the highest across all current densities. YP-50F, in contrast, despite its higher specific surface area than those of RACF-30 and -40, had most of its micropore volume in the pores of ≤1.5 nm diameter, thereby making it difficult to store solvated ions such that the specific capacitance was the lowest and the rate of decrease in specific capacitance was the highest. In addition, the difference in the ion diffusion rate according to the ACF and AC pore structures is presumed to have influenced the rate of decrease in specific capacitance of YP-50F and all of the RACFs.

**Figure 8:**
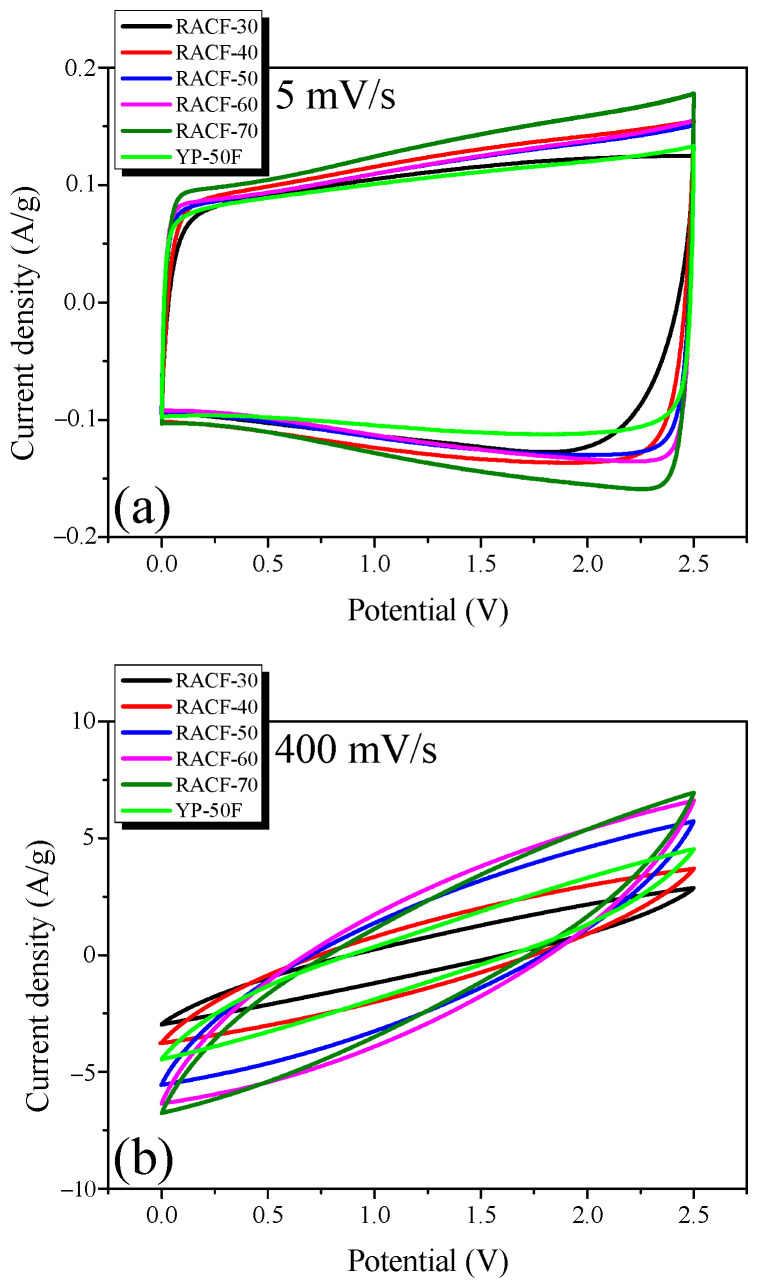
Cyclic voltammograms of RACF at various scan rates: (**a**) 5 mV/s, and (**b**) 400 mV/s.

Cyclic voltammetry was also used since it is a typical means to measure the electrochemical performance of EDLC. Figure 8 shows the CV curves measured at the voltage range of 0–2.5 V and injection rates of 5 and 400 mV/s.

In Figure 8, all CV curves of the RACF samples produce a rectangular shape without evidence of the Faradic redox reaction. In addition, the area of the CV curve that indicates the specific capacitance of the EDLC shows an identical trend to the specific capacitance calculated based on the GCD curve (Table 1). RACF-40, in particular, shows a larger CV curve area than YP-50F, despite its similar specific surface area and low mesopore ratio. This agrees with the previous result from the GCD curve and is presumed to be the case because RACF-40 has a PSD of larger pore diameters than YP-50F. In Figure 8b, however, all CV curves produce a leaf shape rather than a rectangular shape, presumably due to the increase in ion diffusion resistance at the high injection rate of 400 mV/s.

**Figure 9:**
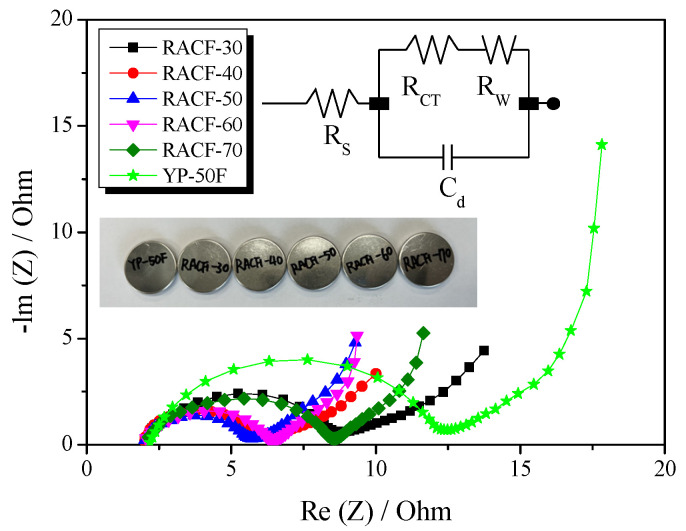
Nyquist plots of RACF obtained using different various activation conditions. The inset shows its equivalent circuit.

Electrochemical impedance spectroscopy (EIS) is a method widely used to analyze the impedance of EDLC and consists of Nyquist plots representing the negative versus imaginary electrochemical impedance of electrodes. Figure 9 presents the Nyquist plots for the RACF, obtained in the frequency range of 10 mHz to 300 kHz through EIS. In addition, the numerical data of the Nyquist plots are given in Table 2. A typical Nyquist plot of an EDLC is generally categorized into three types: bulk solution electrolyte, charge transfer resistance, and Warburg impedance [38]. The first type shows the bulk solution electrolyte resistance (R_S_) that can be easily verified on the x-intercept at high frequency. R_S_ may vary according to the properties of the electrolyte used in the EDLC [38], and in this study, similar values were obtained (R_S_: 2.0–2.2 Ω) across all cells, as can be seen in Table 2, because they were all prepared using 1 M (C_2_H_5_)_4_NBF_4_/PC. The second plot type shows the charge transfer resistance (R_CT_) that is observed in a semicircle shape on the Nyquist plot within the mid-frequency range. R_CT_ is the resistance that incorporates the interface resistance between the active electrode material and the electrolyte, the electrical conductivity of the electrode, the contact resistance between the electrode and the current collector, and the ion resistance of the electrolyte in the pore interior [38]. In this study, RACF showed the smallest value of R_CT_ at 3.7 Ω at 50 min activation time, while the value increased to 6.4 Ω as the activation time increased. In Wang et al. [39], the charge transfer resistance was reported to fall when the electrical conductivity of the EDLC electrode was improved. Thus, the electrical conductivity of RACF in this study is likely to have shown a relative increase in the proportion of crystal grain until 50 min activation time due to amorphous oxidation. Accordingly, the electrical conductivity of the RACF is thus presumed to have increased with amorphous oxidation as the activation time increased, consequently inducing a decrease in R_CT_ in proportion to the activation time. However, as was shown in Figure 4, the edge oxidation of crystal grains after 60 min activation time is likely to have collapsed the micropores towards the expansion of mesopores, leading to a decrease in the electrical conductivity of the RACF. This decrease in the electrical conductivity is presumed to have increased the charge transfer resistance for RACF-60 and -70, promoting the semicircular shape. For YP-50F, a larger value of R_CT_ at 10.2 Ω was observed as compared to all RACF samples. As previously shown, while both the RACF and YP-50F were produced from precursors of identical biomass, the precursor of the RACF was composed of high-purity cellulose with a lower content of hemicellulose than that of YP-50F, having higher crystallinity. Thus, the crystallinity of YP-50F is presumed to be lower than that of the LCAF, leading to lower electrical conductivity than the RACF and hence the highest R_CT_.

The third Nyquist plot type shows the Warburg impedance (R_W_) which is observed as a straight line with 45° slope at low frequency. R_W_ indicates the ion diffusion resistance of the electrolyte [38]. In a prior investigation, the ion diffusion resistance of AC was shown to decrease with an increase in mesopore volume [37]. In this study, the mesopore fraction of the RACF increased to 10.6–31.1% as the activation time increased. Hence, the mesopore fraction increased as a function of activation time, leading to the continuous decrease in R_W_ from 3.63 to 2.23 Ω. Notably, RACF-70 was shown to have the lowest R_W_ according to its high mesopore fraction. YP-50F, despite having a higher mesopore fraction than RACF-40, showed a larger R_W_ at 3.21 Ω. It is generally known that the rate of adsorption is higher for ACF than AC, when the micropores of ACF are open to the exterior. Thus, RACF-40 is thought to have a smaller R_W_ despite its lower mesopore fraction than YP-50F due to its unique pore structure of ACF.

## 3. Materials and Methods

### 3.1. Sample Preparation

Stabilized rayon fiber was obtained from Dissol (Jeonju, Korea) for use as the precursor of the ACF. Commercial AC (YP-50F, Kuraray Chemical Corporation, Tokyo, Japan) was used as the control for assessing the electrochemical properties of the ACF.

The rayon fiber was placed in an alumina boat by 20 g for the carbonization in the cylindrical tubular furnace fabricated in this study (SIC heater: 80 × 800 mm). The heating of rayon fiber reached 700 °C at the rate of 10 °C/min in N_2_ atmosphere, and the carbonization temperature was maintained for 1 h. The carbonization yield of rayon fiber was approximately 54%.

The rayon-based carbon fiber was placed in an alumina boat by 3 g for activation in the same cylindrical tubular furnace. The heating of the rayon-based carbon fiber reached the activation temperature of 900 °C at the rate of 10 °C/min in N_2_ atmosphere (N_2_, 99.999%). Once the activation temperature was achieved, the gas flow inside the reactor was converted to steam at 0.5 mL/min, and the rayon-based carbon fiber was activated for 30–70 min. The sample names for the activated carbon fibers were labeled as RACF-30, -40, -50, -60, and -70, based on the activation time regulations.

### 3.2. Characterization

The textural properties of the RACF samples were measured after 12 h of drying at 573 K and ≤0.133 Pa residual pressure using an isotherm gas adsorption analyzer (BELSORP-max, BEL Japan, Tokyo, Japan). The specific surface area was calculated from the isotherm adsorption curve using the BET (Brunauer–Emmett–Teller) equation [40]. The pore size distribution (PSD) was obtained through the non-localized density functional theory (NLDFT) [41].

The morphologies of RACF were explored using a scanning electron microscope (SEM, AIS2000C, Seron Tech. Inc., Uiwang-si, Korea). The changes in RACF crystalline structure based on activation time were analyzed through X-ray diffraction (XRD) spectroscopy (X-ray diffractometer, X’ pert powder, PANalytical, Almelo, The Netherlands). The XRD pattern was measured using CuKα (1.5406 Å) in 10–60° range at 2°/min. In addition, the RACF crystal grain size (L_a_) and height (L_c_) as a function of activation time were calculated from the XRD pattern using the Gaussian calculation and Scherrer equation [42]:

(1) $$L=\frac{K\lambda }{B\cos\theta }$$

where *λ* indicates the wavelength of the applied X-ray, *B* indicates the full width at half maximum intensity, *K* indicates the dimensionless shape factor L_c_ (0.91) and L_a_ (1.84), and *θ* indicates the Bragg angle.

### 3.3. Electrochemical Measurements

To assess the electrochemical properties of the RACF samples, the electrodes in an EDLC were prepared at the ratio of [active material:conductive agent:binder] = [84:7:9] wt%. The conductive agent was carbon black (Super P, Timcal Ltd., Bodio, Switzerland), and for the binder, carboxy-methylcellulose (Dai-Ichi Kogyo Seiyaku Co., Ltd., Kyoto, Japan), styrene-butadiene rubber (BM400B, Zeon, Tokyo, Japan), and polytetrafluoroethylene (9002-84-0, Sigma Aldrich, St. Louis, MO, USA) were used. The slurry containing the active material, conductive agent, and binder was coated 0.152 mm thick on aluminum foil by the laboratory scale doctor blade coater. The coated electrodes were dried overnight in a 100 °C vacuum oven, then punched to 12 mm diameter. The EDLC was fabricated based on the CR2032 specification with punched electrodes and cellulose-based separation membrane (NKK, Kanagawa, Japan) using 1M (C_2_H_5_)_4_NBF_4_/propylene carbonate organic solution as the electrolyte.

For galvanostatic charge-discharge (GCD) testing of the EDLC, the MACCOR 4300 Battery Tester (Maccor Inc., Tulsa, OK, USA) was used. For cyclic voltammetry (CV) and electrochemical impedance spectroscopy (EIS), a VSP electro-chemical workstation (Bio-Logic Science Instruments, Grenoble, France) was used. The GCD was measured at the voltage range of 0.1–2.5 V and 2–50 mA/cm^2^ current density. The CV was measured at the voltage range of 0.1–2.5 V and 5–400 mV/s injection rate. The frequency range for the Nyquist plots was 10–300 mHz. All electrochemical tests were performed ten times at room temperature, and the data of ten measurements were used in subsequent analyses.

The specific capacitance (capacitance per electrode weight) of the EDLC was calculated using the GCD curve and the weight of the active electrode material based on Equation (2):

(2) $$C_{g}=\frac{i\Delta t}{m\Delta V}$$

where *i* is the discharge current (A), Δ*t* is the discharge time (s), *m* is the mass of the active electrode material, and Δ*V* is the voltage (*V*).

## 4. Conclusions

In this study, RACFs were developed using the steam activation method as a lower-cost and environmentally friendly alternative to nanocarbon materials for enhancing the performance of the EDLC. The effects of various activation times (30 to 70 min) on the ACF micropore structure and pore development behavior were examined; using isotherm adsorption with BET and NLDFT calculations to determine the specific surface area and distribution of pore sizes, XRD to elucidate pore structural changes, and GCD curves, CV, and EIS to evaluate electrochemical performance. The results showed that, with an increase in activation time, the pore characteristics of RACF underwent changes from micropore-rich structure to mesopore-rich structure. Notably, as activation time increased, RACF showed outstanding pore characteristics, including specific surface area of 1990–2150 m^2^/g and mesopore fraction of 20.5–31.1%, despite high activation yield at approximately 28–37%.

The specific capacitance of the active electrode material for EDLC was markedly dependent on the distribution of the pore sizes as a function of activation time. Of the five RACF samples RACF-30 to -70 corresponding to activation times, RACF-40 showed high specific capacitance at approximately 6% despite having a similar specific surface area to that of commercial AC and low total pore volume. Notably, compared to YP-50F at current densities of 2 and 50 mA/cm^2^, the specific capacitance of RACF-70 was shown to have increased by approximately 26% and 150% to 103.6 and 85.8 F/g, respectively. In addition, RACF showed outstanding impedance properties (low IR-drop, low charge transfer resistance, and low Warburg impedance) based on the unique pore structure of its ACF and large diameter of its PSD. This suggests that the pore structure of RACF had been better optimized than that of commercial AC (YP-50F) at 1M (C_2_H_5_)_4_NBF_4_/PC. Overall, the RACF developed through steam activation exhibited more outstanding pore characteristics and electrochemical performance than YP-50F, as well as the potential to enhance the performance of EDLC as an active electrode material. The findings of this study are expected to advance the application of RACF as an effective performance enhancing electrode material for the EDLC, with the advantages of having low cost and being environmentally friendly.

## Figures and Tables

**Table 1:** Textural properties of RACF as a function of various activation conditions.

Sample	S_BET_^a^ (m^2^/g)	V_Total_^b^ (cm^3^/g)	V_Micro_^c^ (cm^3^/g)	V_Meso_^d^ (cm^3^/g)	R_Meso_^e^ (%)	Yield ^f^ (%)	C_g_^g^ (F/g)
RACF-30	1600	0.66	0.59	0.07	10.6	55.0	82.6
RACF-40	1760	0.74	0.65	0.09	12.2	48.9	87.1
RACF-50	1990	0.88	0.70	0.18	20.5	37.2	90.8
RACF-60	2200	1.03	0.74	0.29	28.2	30.7	91.0
RACF-70	2150	1.03	0.71	0.32	31.1	28.0	103.6
YP-50F	1780	0.83	0.70	0.13	15.7	-	82.0

^a^ S_BET_: Specific surface area; BET method $\frac{P}{v\left(P_{0}-P\right)}=\frac{1}{v_{m}c}+\frac{c-1}{v_{m}c}\frac{P}{P_{0}}$. ^b^ V_Total_: Total pore volume; the amount adsorbed P/P_0_ = 0.99. ^c^ V_Micro_: Micropore volume; V_Total_ − V_Meso_. ^d^ V_Meso_: Mesopore volume; BJH method rp = rk + t, (rp = actual radius of the pore, t = thickness of the adsorbed film). ^e^ R_Meso_: Mesopore volume ratio; $\frac{V_{\mathrm{Meso}}}{V_{\mathrm{Total}}}\times 100$. ^f^ Yield: Activation yield; $\frac{Weight\;of\;activated\;sample}{Weight\;of\;carbonized\;sample\;input}\times 100$. ^g^ C_g_: Specific capacitance at 2 mA/cm^2^.

**Table 2:** Values of equivalent circuit parameters from the fitting of the impedance spectra in Figure 7.

Sample	R_S_^a^ (Ω)	R_CT_^b^ (Ω)	R_W_^c^ (Ω)
RACF-30	2.1	6.6	3.63
RACF-40	2.0	4.1	2.67
RACF-50	2.0	3.7	2.53
RACF-60	2.0	4.4	2.24
RACF-70	2.1	6.4	2.23
YP-50F	2.2	10.2	3.21

^a^ R_S_: bulk electrolyte resistance. ^b^ R_CT_: charge transfer resistance. ^c^ R_W_: Warburg impedance.

## Data Availability

The data presented in this study are available on request from the corresponding author.
